# Intertemporal prosocial behavior: a review and research agenda

**DOI:** 10.3389/fpsyg.2024.1359447

**Published:** 2024-04-08

**Authors:** Emil Persson, Gustav Tinghög, Daniel Västfjäll

**Affiliations:** ^1^Department of Management and Engineering, Division of Economics, Linköping University, Linköping, Sweden; ^2^Department of Health, Medicine and Caring Sciences (HMV), The National Center for Priority Setting in Health Care, Linköping University, Linköping, Sweden; ^3^Department of Behavioural Sciences and Learning, Linköping University, Linköping, Sweden; ^4^Decision Research, Eugene, OR, United States

**Keywords:** prosocial behavior, time, intertemporal, cognitive noise, social brain network

## Abstract

Research on intertemporal and prosocial decisions has largely developed in separate strands of literature. However, many of the decisions we make occur at the intersection of these two dimensions (intertemporal and prosocial). Trust is an example, where a decision today is made with the expectation that another person will reciprocate (or betray) later. A new literature is emerging to explore the role of time in these types of situations, where time and social considerations are intertwined. In many cases, time introduces (or magnifies) an element of uncertainty about future outcomes and utility that people need to deal with – what will happen, how good will it be, how will it feel. We review this emerging literature on intertemporal prosocial decision-making and discuss how new research can fill existing knowledge gaps.

## 1 Introduction

Time is a fundamental component in human behavior and interaction. Decisions, desires, views, experiences, and emotions act on and play out over time. Social norms evolve. Societies change. For these reasons, studying how prosocial decisions (here interpreted broadly as decisions affecting and being affected by others) are shaped by time, using controlled experiments, and developing theory, seems important. However, historically this perspective has received little attention. The literatures on intertemporal and prosocial decisions, although each large and vibrant, have mostly developed without significant transfer between them.

Lately, this perplexing isolation between research fields has begun to change. A new literature is emerging at the intersection of temporal and prosocial behavior. What “prosocial” brings to the table here is, primarily, that these decisions often involve a strategic component, where outcomes depend on decisions and expectations by more than one individual; and are to a greater degree influenced by context, norms, personal and social identity. These aspects of decision-making are amenable to time. For example, if people have different motivations for delayed rewards (discounting), then simply adding a time delay to a classic bargaining- or coordination game may substantially change the strategic landscape (and the corresponding game predictions).

Here, we review this emerging literature on intertemporal prosocial decision-making and discuss several promising directions for new research.

## 2 Emerging literature on intertemporal prosocial decision-making

The core research strategy for most papers in this emerging field has been to add a time delay to a standard prosocial decision-context normally run in the lab, e.g., a trust-, cooperation-, coordination-, or bargaining game. This has produced some thought-provoking findings and predictions, including that small differences in time preference may improve coordination and influence bargaining power (about resource distribution), but also that time may act as a barrier to cooperation. Moreover, initial results indicate that trust and trustworthiness is surprisingly robust to time delay, at least for short delays, and that altruism follows a different intertemporal pattern compared to decisions that are purely personal, including absence of classical time-inconsistent choosing.

Agranov et al. (2023) investigated how differential time preferences influenced coordination. They used a standard protocol for repeated coordination, where participants make decisions over many rounds and continuation is probabilistic. Interestingly, allowing for even a small difference in time preference influenced outcomes (fewer coordination failures), likely working as a coordination anchor; and larger differences gave rise to intertemporal trades, where individuals with steeper discounting received higher payoffs early on, and vice versa for individuals with less steep discounting.

Kim et al. (2023) used a similar approach allowing for discounting differentials to investigate the effect on bargaining behavior. In their experiment, participants bargained repeatedly (submitting alternating offers) about how to share a fix sum of money. Any offer that was rejected triggered a new round of bargaining but also pushed eventual payout further into the future. Here, patience is strategically relevant since it is less costly for more patient players to ‘wait’ for a good offer. Theoretically, opposing players who are less patient should recognize and submit better offers upfront. The results showed that players who faced a longer payoff delay (thus steeper effective discounting) indeed submitted less demanding proposals and enjoyed less favorable outcomes overall.

Two papers investigated the effects of time delay on cooperation. Kim (2023) incorporated time delay in payoffs in a repeated prisoner’s dilemma game. They used a classic repeated-games framework but with the temporal sequence of stage games mapped to staggered payouts for weeks or months, rather than all at once received immediately at the end of the experiment. There was lower cooperation when payoffs were delayed more, thus indicating that steeper discounting decreases cooperation in a repeated games framework. Kölle and Laurer (2024) investigated the effects of time delay on various payoff components in a linear public goods game. In the classic version of this game, participants can keep money for themselves or contribute to a common account that benefits everyone in the group. The parameters are usually set such that it is individually optimal for selfish persons to keep all money for themselves, but socially optimal if everyone contributes fully to the group account. Here, the authors systematically varied which type of benefit (personal, group, none, or both) that was paid immediately and which was paid with a 1 year delay. They found overall substantially lower cooperation rates when group benefits were delayed, and, conversely, increased cooperation when personal benefits were delayed instead. Together these studies suggest that time may act as a barrier to cooperation in the context of solving collective problems, like climate change.

Ederer and Schneider (2022) introduced a time dimension in the classic trust game. Unlike the papers surveyed above, here they kept delay to payout constant but instead systematically varied delay to decision. In the trust game, one player (the ‘trustor’) is given a sum of money and decides how much to send to another player, the ‘trustee.’ The amount sent is multiplied and then the trustee decides how much, if anything, to return to the trustor. Using a variant of this game, Ederer and Schneider introduced delay to the trustee’s decision. Participants as trustors thus decided how much to send to their assigned trustees knowing that the trustee would make their decisions either immediately, 1 day after the experiment, or in 3 weeks’ time. They speculate that trustees’ potential feelings of obligation or guilt might decrease as temporal distance (time since trustor’s decision) increases, and as a result, observed trustworthiness and trust should decrease. However, neither trust nor trustworthiness changed substantially for the delays considered in the experiment (max 3 weeks).

Finally, some papers have investigated the temporal dimension of altruistic behavior. Kölle and Wenner (2023) studied how people allocate effort (for payment) for themselves and others inside a time window of 3 weeks, using a task developed to measure time inconsistency (Augenblick et al., 2015). In short, time inconsistency here means disproportionate valuation of rewards that are immediately available. The novel idea here was that people made temporal allocations not only for themselves but also for others and in particular for self vs. other. In line with previous literature (non-social decisions), they found evidence of time inconsistency when choosing for oneself. However, these results did not extend to choices for others or for self-other tradeoffs. The authors concluded that discounting in social situations appears to be conceptually different from personal discounting. Chopra et al. (2024) also investigated the temporal structure of self-other tradeoffs but used a different design, with substantially longer time frames (up to 1 year) and monetary donations to charity. Interestingly, they found that prosocial decisions in the form of self-other tradeoffs had a distinct temporal profile beyond people’s discounting in either domain (self and other when considered separately) and controlling for their atemporal preference for giving in this context. The authors interpreted this as the upshot of a conceptual distinction between temporal utility flows from consequences vs. choices (Kovarik, 2009; Andreoni and Serra-Garcia, 2021 on the role of time in altruistic behavior).

## 3 Recent advances in the intertemporal choice literature

Discounting of future monetary rewards is probably one of the most well-researched topics in behavioral social science, and the literature is very active. Over the years a number of stylized patterns have been documented using experiments, e.g., hyperbolicity (i.e., insensitivity to the length of time intervals) and present bias (i.e., disproportionate valuation of immediate rewards), and different theories have been developed to explain them (see, e.g., review by Cohen et al., 2020). Traditionally, most of these explanations have focused on motivational factors – deep preference for sooner rewards, self-control failures, impulsivity (Enke et al., 2023).

Interestingly, a newer set of papers has emphasized and begun to formalize temporal discounting from a different explanatory perspective, resulting from ‘cognitive noise’ or decision complexity. Gabaix and Laibson (2022) model temporal discounting as resulting from internal uncertainty about value of future events. Decision makers handle this uncertainty by generating noisy mental simulations of future value, which they combine with their prior beliefs. If noise increases with the horizon (time to delivery) this produces a classic discounting pattern, where future rewards are valued less, even for agents who have no real time preference. Vieider (2021) develops a discounting model based on a similar type of cognitive micro-foundation and Bayesian updating approach, except uncertainty here is tied to perception of time delay rather than future utilities. Gershman and Bhui (2020) extend Gabaix and Laibson’s baseline model to account for adaptive simulation noise. Assuming that attention to signals (mental representations of future utility) is costly, people will “think harder” when potential utility is larger and thus appear to adapt their discounting to reward magnitude, which reproduces a stylized discounting pattern known as ‘the magnitude effect.’ Enke et al. (2023) and Enke and Graeber (2023) operate along the same lines but also make a broader methodological point, noting that temporal choices belong to a class of complex decision problems where people may be reluctant, or unable, to engage in the type of cognitive operations required for optimal choice, and rather resort to simpler decision rules. And many of these rules will produce choice patterns that look like classic discounting even when this is not the underlying value guiding people’s choices.

These developments in the intertemporal choice literature may prove useful for understanding how prosocial decisions are made in an intertemporal context. Because both dimensions (intertemporal and prosocial, respectively) can be conceptualized in terms of mental perspective taking, focusing on current vs. future, or self vs. other. This idea is already a conceptual cornerstone for prosocial decisions, where it is important to understand how somebody else will feel, think, and react, and thus well aligned with the idea (echoed by some of the papers reviewed above) that intertemporal choices are influenced by the extent to which people can understand how something will be or play out in the future.

## 4 Research agenda

This new literature on intertemporal prosocial decision-making is just emerging and there are many open questions to address. A first line of inquiry should build on the ideas introduced in the papers written so far, where the focus has been to explore how an added time dimension changes the strategic landscape and what observable effects this may have on behavior. This is an exciting topic where much more research is needed, both to replicate patterns that emerged from the few existing studies and to extend to other contexts.

A second aspect to explore is temporal direction; what happens if decisions are extending into the past compared to into the future? Of course, answering this question will require some elaborate experimental-design work, but it is an important perspective to consider. It applies to most games where decisions (thus not only outcomes) are detached in time. The trust game is an example, where the trustor sees to the future but the trustee sees to the past. Conceptually it is not evident that the past and the future are symmetrically perceived. For instance, whereas the present can be experienced, the past and the future have to be mentally constructed (Trope and Liberman, 2010). The source of these construals will be based on memory and imagination (prediction), the past, arguably, to a greater extent on memory, and the future on prediction (Schacter et al., 2017). Down the line there will be interesting connections to be made with literatures on motivated memory and experience-based prediction.

A third, more fundamental task is to start building a conceptual home ground for prosocial decision-making in a temporal context. There is currently no established, unifying conceptual framework. This is not surprising, given that both temporal and prosocial decision-making are complex matters on their own, with a host of different theories existing in either domain, and these theories often rest on different psychological and neural foundations. However, eventually we need to break new ground here. A valuable first step in this process would be to shift focus a little bit by starting to collect large amounts of data within subjects, with the goal of building, and eventually estimating, temporal profiles of prosocial behavior in different contexts. The blueprint would be a densely populated (many different delays) delay discounting task adapted to prosocial choices. These profiles would then form the basis for exploring the effects of different experimental manipulations or looking for differences related to quantitative trait.

Going forward, one possible way to bridge the divide between prior separate conceptualizations of prosocial and temporal choice is to look to new conceptual work in the intertemporal choice literature (reviewed in short above), which emphasizes the role of noisy cognition (and decision complexity) in temporal discounting. One interesting line of thinking here is that decision makers may try to resolve uncertainty about the future by using mental simulations (what will happen, how good will it be, how will I feel), and that this results in temporal discounting. The focus on mental perspective-taking (mental simulations) for temporal decisions is key here because it is conceptually well aligned with the type of cognitive processes we typically attribute to prosocial decisions – “mentalizing” about what we believe other persons will do, how they will feel, what they expect from us, and so on (Chang et al., 2023). This suggests a basic hypothesis about shared substrates in mental perspective taking that are relevant for prosocial decision-making in an intertemporal context. There are many ways in which new research could seek more evidence for (or refute) this hypothesis. One way would be to systematically manipulate temporal distance using episodic time cues (Peters and Büchel, 2010) or emotional salience, e.g., fear of betrayal in the trust game, which should both have a predictable temporal component in their influence on behavior. Ideally this approach is then combined with a search for underlying neural mechanisms, which we touch on briefly below.

An emerging hypothesis in decision neuroscience is that overlapping brain networks are involved in both prosocial and intertemporal decisions. Correlational studies using functional magnetic resonance imaging (fMRI) have shown that a specific set of brain areas are consistently activated for prosocial decisions, often referred to as the social brain network (or the mentalizing network) (Alós-Ferrer and Farolfi, 2019; Chang et al., 2023). A key functionality facilitated by this network is the cognitive ability to understand and predict other persons’ intentions, beliefs, and actions; a form of mental perspective taking that is crucial for beneficial social interaction. Of note, a similar type of functionality (using a shared neural implementation) is thought to be operating also in temporal decisions (Soutschek et al., 2016). Here the hypothesis is that the mentalizing network enables future perspective taking, thus influencing the extent to which the future is valued, from today’s perspective. This points to a novel mechanism that is quite different from classic temptation/self-control models of intertemporal choice.

Current thinking is that the regions involved in prosocial or temporal decision-making (e.g., the temporoparietal junction or the partially overlapping angular gyrus) provide higher-order cognitive functions that facilitate integration of multisensory input, and that this functionality is particularly useful when building mental representations of complex phenomena, like social context or temporal projection (Jung et al., 2022; Humphreys and Tibon, 2023; Lugrin et al., 2023). Consistent with this conceptualization, Pietrzak et al. (2023) found that neural activity in angular gyrus and surrounding areas correlated with decisions in a standard temporal discounting task. An interesting and important avenue for future research is thus to establish the casual role (and connectivity) of key regions in the mentalizing network for prosocial decisions made in a temporal context.

## 5 Discussion

An exciting new literature is emerging at the intersection of temporal and prosocial decision-making. Our review highlighted directions this literature is taking and we discussed knowledge gaps to be filled by future research. Most of our focus was on the need for in-depth understanding of decisions and underlying processes, including unifying conceptualization. Ultimately such deeper understanding will be needed for extrapolating findings to the world outside the experimental lab. And this is where this new literature becomes relevant for real – because the intertemporal prosocial dimension is present in some of today’s biggest societal challenges. How to find behavioral solutions that can speed up climate change mitigation is perhaps the clearest example. Time is important here because costs will be borne now and benefits in the future. Same for health policy, which discounts the lives of future generations. And both of these decisions are made in a social context.

In the behavioral social-science literature, bottom-up climate change mitigation is often cast in a cooperation/social-dilemma type of framework. Here, the key problem for mitigation is the tension that exists between individually optimal behavior (selfishness) and socially optimal behavior (full cooperation). An important question for policy is therefore how to make people more cooperative, and when it comes to environment this often means increase willingness to bear personal costs of climate-friendly actions incurred today (e.g., price, effort, comfort) for common benefits at some point in the future. As noted by the few papers we surveyed on this topic above, time is important to consider here because willingness to cooperate is plausibly influenced by when the benefit (e.g., global warming kept below 1.5°C) is expected to materialize, or when everyone else make their decisions (e.g., overexploitation is often temporally detached among different actors). More research here can help us design better interventions to tackle these problems.

Of course, climate action is not the only topic where understanding the role of time is important. Motivated beliefs, teamwork, trust, economic hold-up are other examples where social outcomes are plausibly shaped by time. We anticipate new exciting research on these and other topics over the coming years, as the emerging literature on intertemporal prosocial choice continues to grow.

For policymaking, the question how to think about time in prosocial decision-making is not only relevant from a behavioral-descriptive point of view; it is also a highly normative matter, which has consequences for the well-being of both current and future generations. Increased knowledge about when and why time shapes behavior and preferences for policy is just a first step when discussing the more fundamental question of when and why time preferences *should* shape behavior and public policy. How prosocial choices are shaped by time in an intergenerational context is also a question of utmost importance not only for future generations but for the structure of governance if modern democracy as a mechanism for public decision-making is insufficiently sensitive to the concerns of future generations.

## Author contributions

EP: Writing – original draft, Conceptualization. GT: Writing – review & editing, Conceptualization. DV: Writing – review & editing, Conceptualization.
